# Urine oxygenation predicts COVID-19 risk

**DOI:** 10.1007/s10157-023-02456-5

**Published:** 2024-02-24

**Authors:** Eisei Noiri, Daisuke Katagiri, Yusuke Asai, Takeshi Sugaya, Katsushi Tokunaga

**Affiliations:** 1 National Center Biobank Network (NCBN), Central Biobank, National Center for Global Health and Medicine (NCGM), 1-21-1 Toyama, Shinjuku, Tokyo, 162-8655 Japan; 2 Department of Nephrology, National Center for Global Health and Medicine, Tokyo, Japan; 3 Antimicrobial Resistance Clinical Reference Center, Disease Control and Prevention Center, National Center for Global Health and Medicine (NCGM), Tokyo, Japan; 4 Nephrology, St Marianna University, Kawasaki, Japan

**Keywords:** L-type fatty acid-binding protein (L-FABP), FABP1, Urine, Acute respiratory distress syndrome (ARDS), Systemic inflammatory response syndrome (SIRS)

## Abstract

Since February, 2023, the omicron variant has accounted for essentially all new coronavirus infections in Japan. If future infections involve mutant strains with the same level of infectivity and virulence as omicron, the government’s basic policy will be to prevent the spread of infection, without compromising socioeconomic activities. Objectives include protecting pregnant women and elderly persons, and focusing on citizens requiring hospitalization and those at risk of serious illness, without imposing new social restrictions. Although the government tries to raise public awareness through education, most people affected by COVID-19 stay at home, and by the time patients become aware of the seriousness of their disease, it has often reached moderate or higher severity. In this review, we discuss why this situation persists even though the disease seems to have become milder with the shift from the delta variant to omicron. We also propose a pathophysiological method to determine the risk of severe illness. This assessment can be made at home in the early stages of COVID-19 infection, using urine analysis. Applicability of this method to drug discovery and development is also discussed.

## Introduction

Three years have passed since the COVID-19 outbreak in Wuhan, China, which spread throughout the world, and became entirely too familiar to people everywhere. Fortunately, compared to the early days of the pandemic, when treatment of this infection was nonexistent, it is now possible in developed countries to manage and treat moderate and severe cases with therapeutants and medical equipment. The virus has evolved through several variants, initially becoming progressively more virulent and infectious, until omicron, arguably the most contagious of all, but fortunately less serious [1, 2]. This has decreased the frequency of severe cases, although the population remains susceptible to infection. The number of infections leading to severe cases, hospitalization and death, has decreased since the days of the delta variant [3]. Even so, deaths occasionally occur while patients are recovering at home. Sometimes these deaths are solitary, but often problems arise because emergency medical teams are unable to find hospitals that can accept such patients, even though compared to the delta variant era, COVID-19 critical patients currently occupy fewer hospital beds in urban areas [4–6].

Why do such problems occur? Regardless of hospital size, infection control is obviously of paramount importance for both medical care and hospital management. In the case of highly contagious COVID-19 infections, horizontal transmission is facile and infection control is difficult. Therefore, infection cycles often occur between hospitalized patients and medical workers, leading to institutional clusters of cases. Once a cluster is identified, patient admissions to that hospital are restricted by various means. Therefore, because COVID-19 outbreaks restrict access to hospitals, lives that could have been saved before the pandemic may not be saved now, though this is not to suggest that infection control should be neglected.

### Why urinalysis is appropriate?

In the beta and delta stages of the pandemic, COVID-19-positive patients assigned to home rest, lived in fear that they might become severely ill. Now, if COVID-19-positive subjects who are at risk of serious illness can be identified during early stages of infection, morbidity, mortality, and social disruption can be prevented. Early detection of those at risk for severe illness is like searching for pebbles in an ocean, because the overwhelming majority of people remain mildly ill or asymptomatic.

Since the time of Hippocrates, urine has proven a very useful and non-invasive indicator of the condition of the whole body. The practice became so divinatory that its use eventually fell into disrepute after the Middle Ages. However, with the invention of the microscope, diagnosis of urinary sediment became possible, and urine stick tests enabled qualitative identification of proteinuria, urinary sugar, urinary occult blood, and nitrite. These assays are now used in daily medical practice. In Japan, urinary screening is a mandatory part of annual school and workplace health examinations, so the public is well familiar with the ease of urine screening. Such tests are non-invasive and can be used to screen a large number of subjects in a short time, requiring no special skill for stick tests or the equivalent. Moreover, since urine collection is self-administered, there is less risk of transferring infections to others than with blood collection. Nonetheless, coronavirus, which has a molecular mass of 150–200 kDa, does not appear in urine in mild cases [7]. When testing urine for L-FABP, we initially performed urine nucleic acid amplification tests (NAAT) on 200 COVID-19-positive patients to evaluate the possibility of environmental contamination by COVID-19 urine, but all 200 mild to severe cases were negative, soon after diagnosis. Just as urinalysis is useful to identify diabetes mellitus by detecting urinary sugar or useful to identify kidney disease by detecting proteinuria in medical checkups of mostly normal, healthy subjects, likewise, urinalysis makes it easy to distinguish persons at risk of moderate or severe COVID-19 from those who are likely to remain mild or asymptomatic.

### Suitability of urinary L-FABP for risk assessment of COVID-19 infection

Given the global spread of novel coronavirus infections, we are investigating and proposing a novel urinary assay to predict the severity of COVID-19. This research is still ongoing, but among multiple urinary biomarkers, in this review we will focus on urinary L-type fatty acid-binding protein (L-FABP, FABP1). In Europe and Japan, L-FABP has already been approved as an in vitro product to diagnose renal injury [8].

A unique hypoxic condition called “happy hypoxia” appears in moderate or severe cases of coronavirus infection. For this reason, many countries have dealt with this problem by measuring SpO_2_ using pulse oximeters to identify COVID-19-positive patients who require serious interventions. In Japan, among other countries, if patient SpO_2_ values worsen, patients report this and receive intensive treatment through hospitalization. However, SpO_2_ values are prone to fluctuations, especially as the pulse wave becomes weaker with lower oxygenation, leading to unstable values. Sixty torr of PaO_2_ is considered equivalent to 96% SpO_2_ in room air, which is a safety threshold for starting nasal oxygen administration. However, it is difficult for lay persons to determine what action to take if SpO_2_ is oscillating from 93 to 96 to 94, and sometimes the decision to seek hospitalization comes too late. What would happen if we apply the urine biomarker L-FABP to determine the course of action? Below, we will explain how we came to think that L-FABP might work as a urine oxygenation biomarker for COVID-19 patients and the molecular biological mechanism by which L-FABP reflects systemic oxygenation.

## Method for the proof of concept

COVID-19 was introduced to Japan as the Wuhan-1 variant. The disease broke out during a cruise on the Diamond Princess, a luxury liner from Hong Kong, which was forced to make port in Yokohama. The incident attracted worldwide attention because of a series of horizontal infections on the ship, some of which became severe. The National Center for Global Health and Medicine (NCGM) was the first Japanese hospital to admit infected patients in Japan. At that time, all COVID-19-positive patients in Japan had to be managed at a limited number of hospitals designated by the government. The data presented here come from the first 45 cases sent to the NCGM. On board the passenger ship, patients who tested positive for COVID-19 in RT-PCR tests on consecutive days for suspected horizontal infection were transferred to NCGM. Vital signs necessary for follow-up and disease monitoring included temperature, blood pressure, pulse, SpO2, blood counts and blood biochemistry tests, and urinalysis.

Deterioration of oxygenation can be seen in patient respiratory management as an increase in the fraction of inspiratory oxygen (FiO_2_), i.e., ΔFiO_2_. We examined whether SpO_2_ and other indicators at the time of admission could capture this increase during that one-week period. The maximum value of ΔFiO_2_ was taken as the maximum value during a week. A chest X-ray was performed on the day of admission. In cases with elevated ΔFiO_2_, the patient is at increased risk of developing pneumonia or requiring severe respiratory management. We classified respiratory symptom severity into four groups: (1) no change; (2) COVID-19-related pneumonia, but no need of oxygen therapy; (3) COVID-19-related pneumonia with need of oxygen therapy; and (4) COVID-19-related pneumonia with need of mechanical ventilation therapy. While keeping these as surrogate markers of severe outcomes, urinary L-FABP was evaluated for proof of concept in two groups, with a cut-off of 10 ng/mL, which is also the upper limit for healthy normal individuals.

### Initial clinical data analysis for proof of concept

**Fig. 1:**
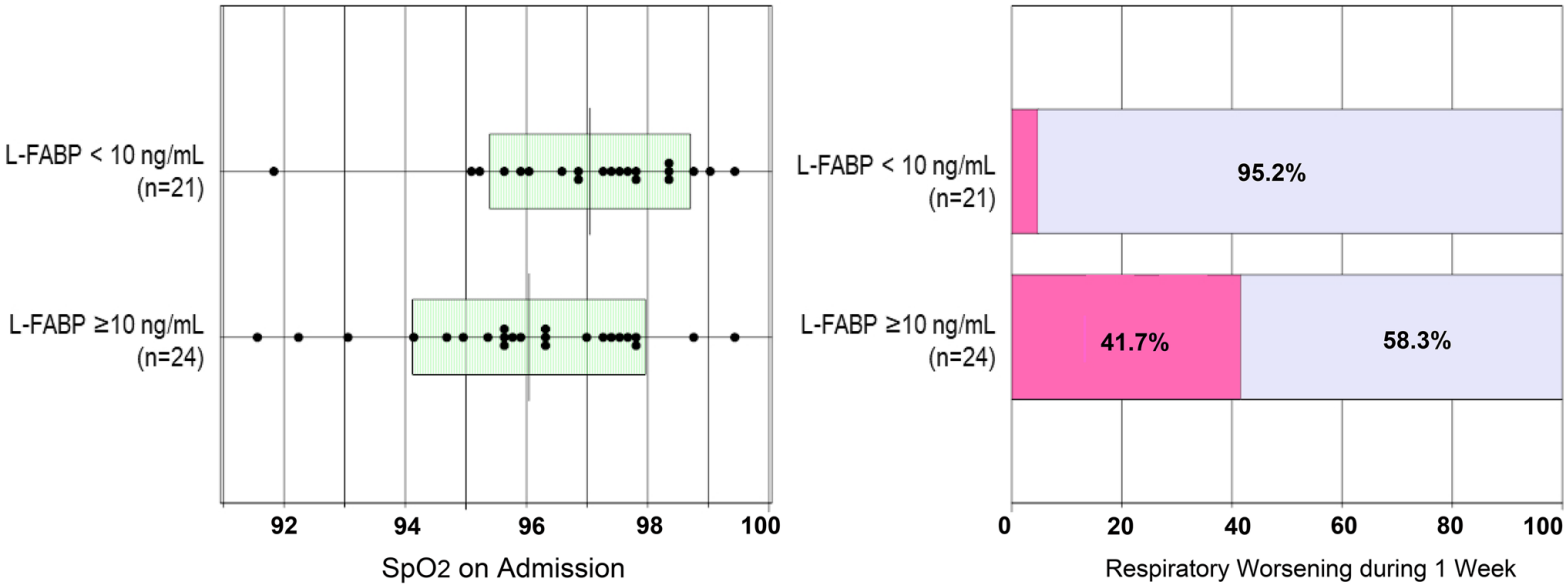
SpO_2_ level at COVID-19 diagnosis and maximum ΔFiO_2_ one week thereafter. Left: Patients were classified into two groups based on a net L-FABP value (10 ng/mL), and SpO_2_ values in the horizontal axis were plotted individually upon admission. The median value was 97% for patients above 10, and 96% for patients below 10. There was statistical difference between two groups, *p* < 0.05 using *t*-test. Right: The proportion of patients who required additional oxygenation after admission was demonstrated through the evaluation of the maximum ΔFiO_2_ increment (FiO_2_ increase) within 1 week after admission. Magenta color indicates the percentage of cases requiring more oxygen after admission for respiratory management. Chi-square detected significant difference between two groups (*p* < 1 × 10^–9^)

**Fig. 2:**
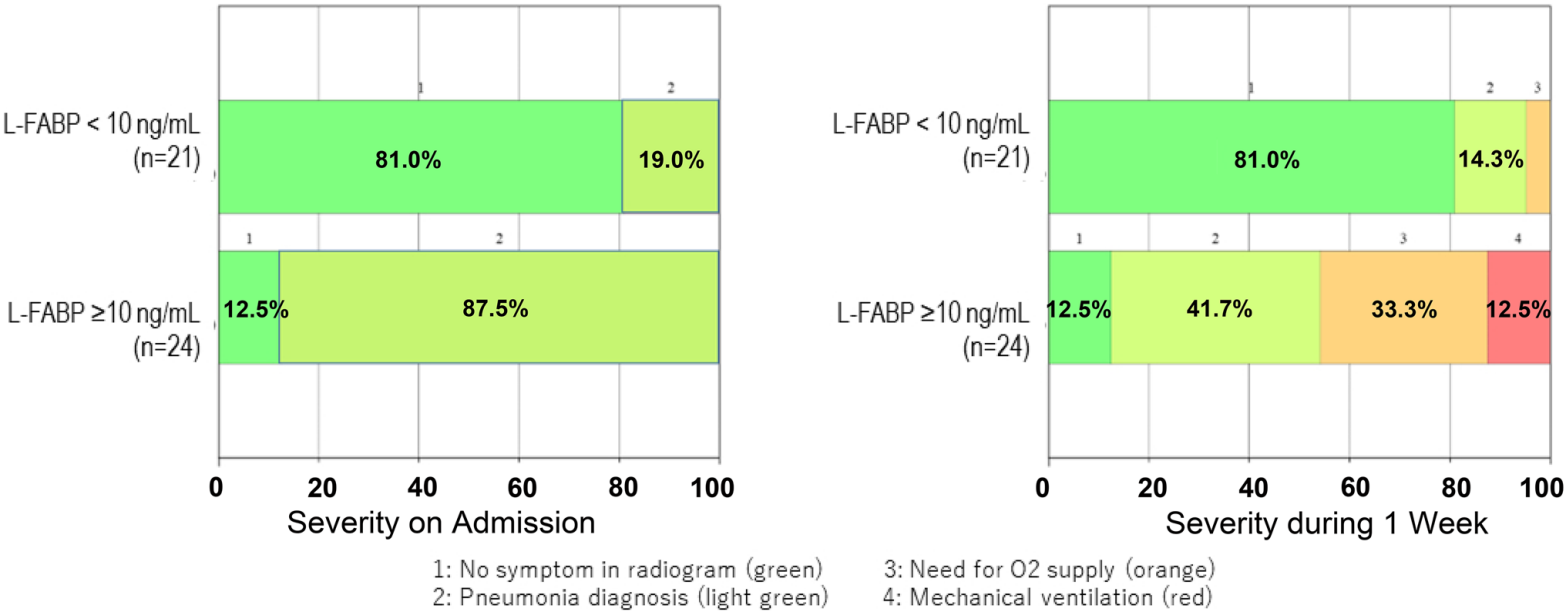
Respiratory severity with or without pneumonia at the time of COVID-19 diagnosis and one week thereafter. Left: Patients were classified into two groups based on a net L-FABP value of 10 ng/mL, and the percentage of patients with or without pneumonia by chest X-ray. Chi-square found significant differences in the presence of pneumonia on admission between two groups (*p* < 1 × 10^–21^). Right: Respiratory severity class percentages 1 week after admission. The severity classification is as follows: 1. No symptoms in radiogram (green), 2. Pneumonia diagnosed by chest X-ray (light green), 3. Oxygen supply needed (orange), 4. Respiratory mechanical ventilation required (red). Colors indicate disease severity. The most severe condition in a week was assigned in each case. Chi-square found statistical differences between groups (*p* < 1 × 10^–20^) and further found the oxygen requirement in higher L-FABP group (ratio of orange and red; *p* < 1 × 10^–10^)

In the aforementioned procedures, the indicator that could have predicted an increase in ΔFiO_2_ and the severity of respiratory management during the first week of onset was L-FABP in the urine (Figs. 1, 2). Urinary L-FABP was classified into two categories with a cut-off of 10 ng/mL (Fig. 1), and data were plotted individually based on SpO_2_ values (horizontal axis). The line shows median values of SpO_2_ in the two groups, with a median value of 97% in the group below 10 and 96% in the group more than 10. The right panel of Fig. 1 shows whether additional oxygenation was required after admission based on the maximum ΔFiO_2_ increment within 1 week after hospitalization. Magenta demonstrates the ratio of patients required more oxygen after admission for respiratory management. Some patients required intubation for ventilator management. Since a 1% variation in SpO_2_ is within the margin of error for the SpO_2_ measurement method, it is difficult to determine a definitive risk of respiratory worsening within a week. On the other hand, the urine L-FABP test is able to detect this risk.

Urinary L-FABP was used to allocate patients into two groups, as described above (Fig. 2). The right panel of Fig. 2 represents the worst of the four conditions above within the first week after admission. There are many cases of severe disease among those with L-FABP > 10 ng/mL. This small, preliminarily study shows that, surprisingly, L-FABP 10 ng/mL, which is almost the upper limit of the normal range used in Europe and Japan to detect renal damage, indicates a patient’s risk of severe COVID 1 week after a positive diagnosis.

### Sensitivity and specificity of risk assessment by urine L-FABP

**Fig. 3:**
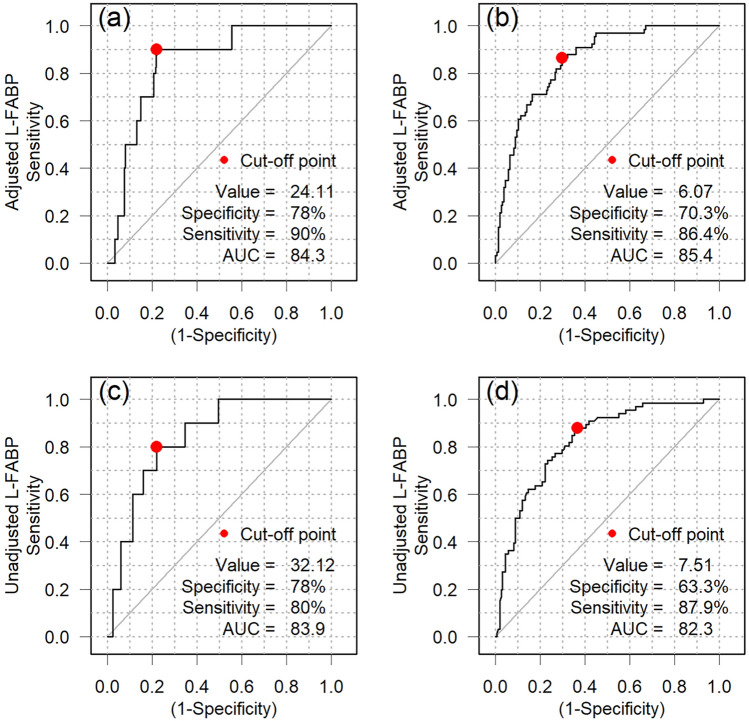
ROC curves to detect severe or mild COVID-19 cases (All cases: *n* = 224). **a**–**d**, ROC curve analysis was performed for all cases (*n* = 224) to detect severe or mild groups, using either adjusted or unadjusted urinary L-FABP levels. Resulting cut-off values were; adjusted L-FABP to discriminate severe cases **a** 24.1 μg/gCre (specificity 78.0% and sensitivity 90.0%, area under the receiver operating characteristic curve [AUC] 84.3%) and mild cases **b** 6.1 μg/gCre (specificity 70.3% and sensitivity 86.4%, AUC 85.4%), and unadjusted L-FABP to discriminate severe cases **c** 32.1 ng/mL (specificity 78.0% and sensitivity 80.0%, AUC 83.9%), and mild cases **d** 7.51 ng/mL (specificity 63.3% and sensitivity 87.9%, AUC 82.3%)

Based on the above preliminary data, in a previous report [9], we reported the usefulness of this method in 58 COVID-19-positive patients. However, the number of patients needs to be increased and the results reconfirmed before such a novel approach can be used in clinical practice. A prospective study was conducted at a couple of centers in Japan, and 522 patients were enrolled [10]. Of 224 cases that met inclusion criteria (patients over 18 years old, without end-stage renal disease, and with urinalysis data during hospitalization) there were 173 mild cases and 51 moderately severe cases. Analysis of patient specimens occurred within 10 days of COVID-19 onset, and within 4 days of hospitalization, including weekend admissions. The AUC of adjusted L-FABP predicting severe outcomes was 84.3% (95% CI 73.5–92.0%) with a cut-off of 24.1 (Fig. 3). Sensitivity was 90.0% and specificity was 78.0%. The AUC of unadjusted L-FABP predicting severe outcomes was 83.9% (95% CI 73.4–92.7%) with a cut-off of 32.1. Sensitivity was 80.0% and specificity was 78.0%. The AUC of adjusted L-FABP predicting mild outcomes was 85.4% (95% CI 79.8–90.3%) with a cut-off of 6.07. Sensitivity was 86.4% and specificity was 70.3%. The AUC of non-adjusted L-FABP predicting mild outcomes was 82.3% (95% CI 76.3–87.7%) with a cut-off 7.51. Sensitivity was 87.9% and specificity was 63.3%. In this study, only 7 patients showed acute kidney injury (AKI) on admission. In other patients, serum creatinine generally remained below 1 mg/dL. Kidney dysfunction was minimal among the current cases.

The public wants severe cases to be detected at infection onset, when symptoms are mild, since persons with mild cases remain at home without being admitted to the hospital. For this purpose, information from our recent study, in which we prospectively examined mild cases and looked at changes in severity, is very useful. Before examining the data, a few characteristics of urinary data require explanation. The water volume of urine changes constantly to maintain an appropriate intravascular water balance. Therefore, it is better to adjust urine solute values by the urinary creatinine value to enhance measurement accuracy.

**Fig. 4:**
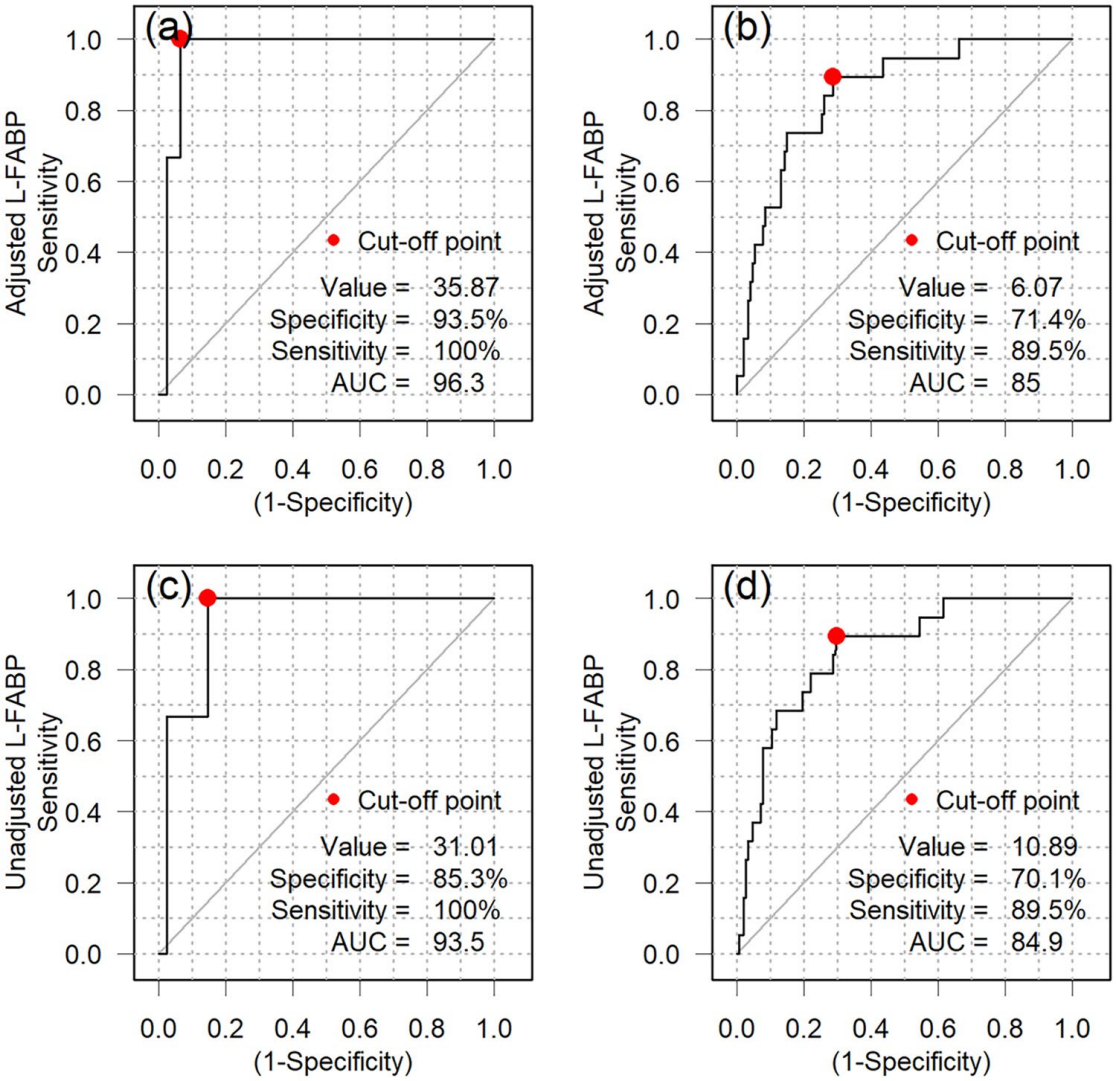
ROC curves to detect severe or mild cases of COVID-19 of initially mild cases. **a**–**d** ROC curve analysis was performed for mild cases at diagnosis of COVID-19 (*n* = 173) to detect severe or mild groups using adjusted urinary L-FABP. Resulting cut-off values were; severe cases **a** 35.9 μg/gCre (specificity 93.6% and sensitivity 100%, AUC 96.3%), mild cases, **b**: 6.1 μg/gCre (specificity 71.4% and sensitivity 89.5%, AUC 85.0%). ROC curve analysis to detect severe or mild groups using adjusted urinary L-FABP. Resulting cut-off values were; severe cases **c** 31.0 ng/mL (specificity 85.3% and sensitivity 100%, AUC 93.6%), mild cases **d** 10.9 ng/mL (specificity 70.1% and sensitivity 89.5%, AUC 84.9%)

Of 173 initially mild cases, 3 became severe and 154 remained mild (Fig. 4). The AUC of L-FABP adjusted for urine creatinine to predict severe outcomes was 96.3% (95% CI 92.6–98.8%) with a cut-off value 35.9. Sensitivity was 100% and specificity was 93.5% (Fig. 4a). This is extremely good predictive capacity. Data without adjustment by urinary creatinine are shown below.

The AUC of L-FABP (unadjusted for urine creatinine) for predicting severe outcome was 93.5% (95% CI 85.3–98.8%) with a cut-off value of 31.0. Sensitivity was 100% and specificity was 85.3% (Fig. 4b). The fact that the results are quite good even without adjustment suggests that the point-of-care (POC) approach may also show good predictive power. Next, we extend the framework for predicting severity of illness to find moderate or severe cases as well.

Among the 173 mild cases, 19 developed into either moderate or severe cases (Fig. 4c). The AUC of L-FABP adjusted for urine creatinine for predicting moderate or severe outcomes was 85.0% (95% CI 75.9–92.3%) with a cut-off 6.07. Sensitivity was 89.5% and specificity was 71.4% (Fig. 2b). The AUC of L-FABP (unadjusted for urine creatinine) for predicting moderate or severe outcomes was 84.9% (95% CI 75.3–92.5%) with a cut-off of 10.9. Sensitivity was 89.5% and specificity was 70.1% (Fig. 4d). Although inclusion of moderate outcomes reduces the predictive capacity, it might be safer to include broader coverage (moderate or severe outcome) in risk management. In the POC operation, the cut-off value of unadjusted L-FABP was 31.0 for severe outcomes only, while it was 6.7 for moderate or severe outcome, suggesting that semi-quantitative evaluation could be used.

### How can urinary L-FABP predict worsening respiratory status?

Urine L-FABP has been approved by the Pharmaceuticals and Medical Devices Agency (PMDA) and CE as an in vitro diagnostic agent that can assist in early diagnosis of renal injury. Its pathophysiology has been extensively studied in acute kidney injury in mice. In rodent kidney, the so-called silencing sequence upstream, nucleotides − 4000 to − 597, of the L-FABP gene are expressed and repress gene expression (11), whereas in humans that sequence is absent and the gene is expressed. Previous L-FABP-related mouse studies employed humanized mice, in which the entire length of the mouse L-FABP gene, including the silencing sequence, was replaced with the human L-FABP gene [12]. The assay system also used a monoclonal antibody that recognizes human L-FABP only and was an ELISA method approved for in vitro diagnostic use.

**Fig. 5:**
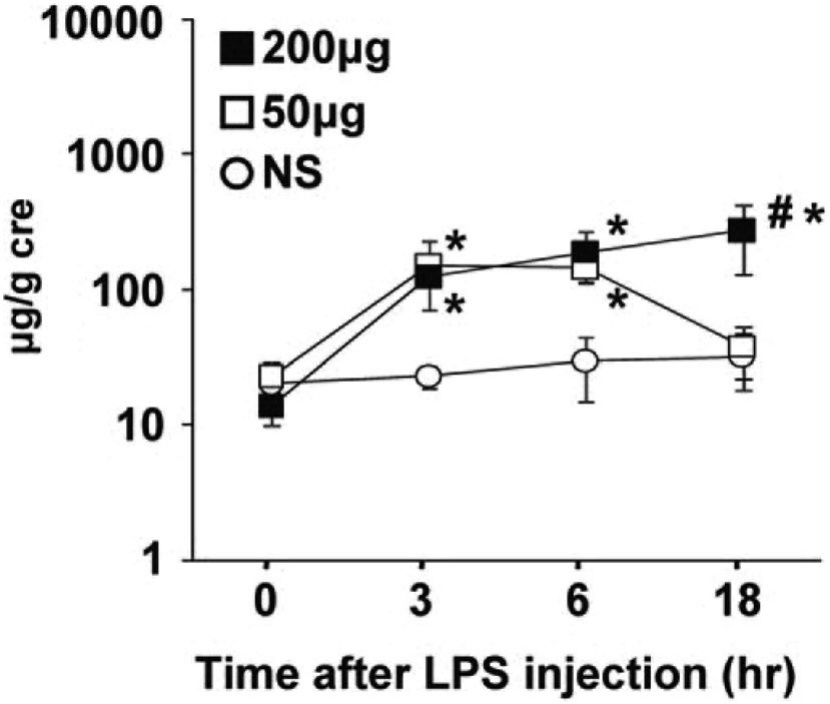
Urinary L-FABP in a mouse intratracheal LPS injection model^16^**.** Bronchoalveolar lavage fluid protein levels differ between the 50 µg and 200 µg group (*n* = 5–7/group, *p* < 0.05 vs. saline injection), denoting the difference of severity

We have reported many studies of AKI, including ischemic injury. Urinary L-FABP proved to be a highly sensitive, specific biomarker for ischemic damage to the kidneys [13–15]. The kidneys are well vascularized organs that receive 20% of cardiac output and that control systemic water and solute balance. Erythropoietin is produced by peritubular capillary endothelial cells in the kidney and serves as a hypoxia sensor. Insufficient oxygenation causes an increase in its expression, leading to an increase in red blood cells and improved systemic oxygenation. Hypoxia sensing depends on a molecular structure called the hypoxia responding element (HRE), upstream of the gene. The L-FABP gene also has an HRE upstream and L-FABP gene expression is also increased in response to hypoxia. This means that even hypoxic conditions that do not result in acute kidney injury are likely to increase urinary L-FABP levels. For proof of concept, we investigated a hypoxic model in which lipopolysaccharides (LPS) were administered to lungs of mice via bronchial tubes to induce hypoxia resulting from a consequent cytokine storm in the lungs [16]. Urinary L-FABP increased in a dose-dependent manner (Fig. 5). A small dose of LPS elevated it transiently, and it soon returned thereafter to the level of control saline-treated mice. In severe COVID-19 patients, the condition known as “happy hypoxia” results from lung-only injury and is very similar to the current mouse LPS model. For this reason, we hypothesized that urinary L-FABP could predict the risk of severe COVID-19 in the absence of acute kidney injury.

### Comparison of urine L-FABP and serum IL-6

In severe COVID-19 cases, immune overreaction is associated with worsening of the disease, and an increase in IL-6 is particularly well correlated with disease severity. This led to development of tocilizumab, a humanized anti-human IL-6 receptor monoclonal antibody, as a potential therapeutic agent. Although initial clinical studies did not demonstrate efficacy, the RECOVERY trial in Japan showed a significantly lower 28 day mortality rate in the tocilizumab group (RR 0.85, 95% CI 0.76–0.94, *p* = 0.0028), particularly in combination with steroid administration. A subsequent meta-analysis of 27 RCT studies was reported in July 2021 with similar results [17]. Serum IL-6 has been approved by the FDA and PMDA as an in vitro diagnostic agent because it is an indicator of disease activity.

**Fig. 6:**
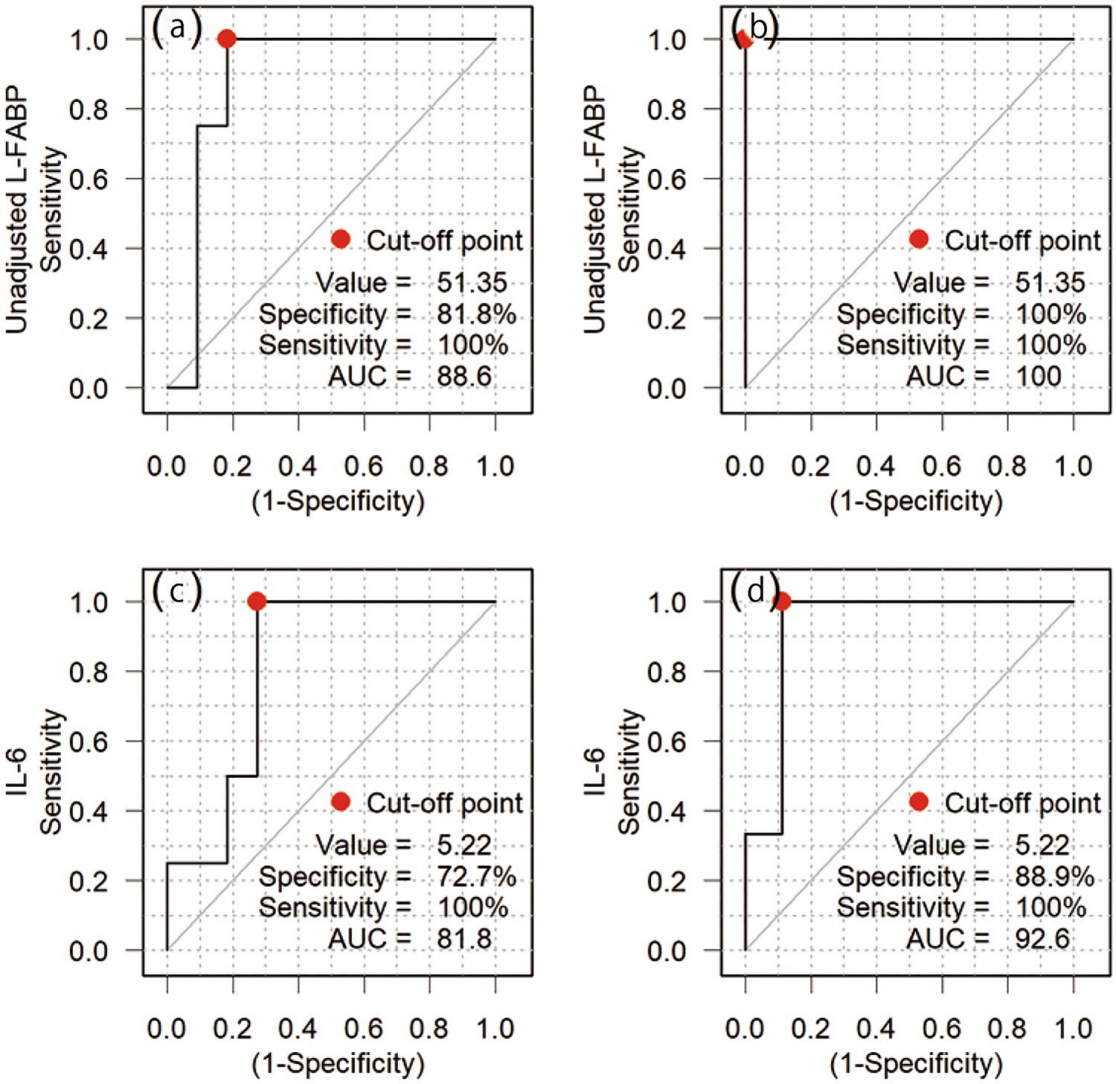
Correlation analysis and ROC analysis to detect severe or mild COVID-19 between urine unadjusted L-FABP and serum IL-6. **a**–**d** ROC curve analysis was performed to detect severe or mild cases using unadjusted urinary L-FABP or serum IL-6. Resulting cut-off values were unadjusted L-FABP; severe cases **a** 51.4 ng/mL (specificity 81.8% and sensitivity 100%, area under the receiver operating characteristic curve [AUC] 88.6%), mild cases **b** 51.4 ng/mL (specificity 100% and sensitivity 100%, AUC 100%), and IL-6; severe cases **c** 5.2 pg/mL (specificity 72.7% and sensitivity 100%, AUC 81.8%), mild cases **d** 5.2 pg/mL (specificity 88.9% and sensitivity 100%, AUC 92.6%)

We argue that urine L-FABP can reveal severe illness trajectory in patients who test positive for COVID-19. How does this compare to serum IL-6? Serum IL-6 was not routinely measured in all cases. In a preliminary study, we examined the correlation between serum IL-6 and urinary L-FABP in cases where they could be measured on the same day. We found that urinary L-FABP correlated very well with serum IL-6 (*r* = 0.78, *p* < 0.001, *n* = 15). Although the number of cases used here was small, ROC analysis showed the following. Among these 15 cases, the AUC of unadjusted L-FABP for predicting severe outcomes was 88.6% with a cut-off of 51.4 ng/dL. Sensitivity was 100% and specificity was 81.8% (Fig. 6a). Six cases progressed to either moderate or severe disease, whereas nine remained mild. The AUC of unadjusted L-FABP for predicting moderate or severe outcomes was 100% with a cut-off 51.4. Sensitivity was 100% and specificity was 100% (Fig. 6b). This suggests that the L-FABP POC test will show good predictive performance in this small cohort. In contrast, the AUC of IL-6 for predicting severe outcomes was 81.8% with a cut-off of 5.2 pg/mL. Sensitivity was 100% and specificity was 72.7% (Fig. 6c). The AUC of IL-6 in predicting moderate or severe outcomes was 92.6% with a cut-off 5.2. Sensitivity was 100% and specificity was 88.9% (Fig. 6d). Since the ROC analysis of L-FABP was comparable to that of IL-6, we decided to plot the measurements versus time for each case with multiple measurement points during the course of hospitalization.

**Fig. 7:**
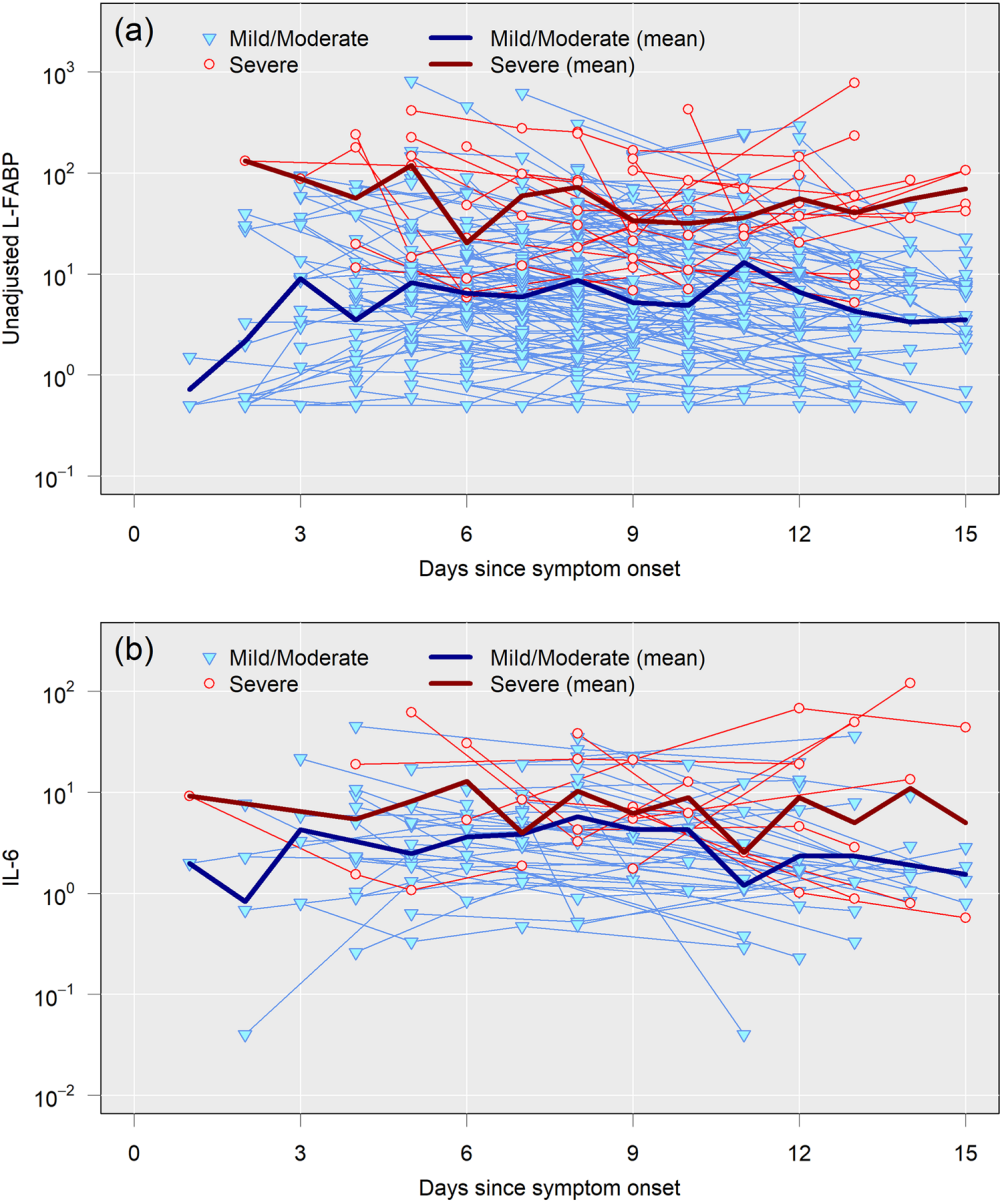
Dynamics of L-FABP and IL-6 during the first 15 days after COVID-19 onset. Urine L-FABP levels (**a** upper panel) and serum IL-6 levels (**b** lower panel) are shown using all cases from NCGM; L-FABP, 228 cases and IL-6, 60 cases. The thick maroon line represents the mean of severe cases. The thick blue line denotes the mean of moderate and mild cases. A wider space between the two lines (maroon and blue), represents a greater likelihood of discriminating severe cases. Mixed-effect model was used to compare L-FABP and IL-6. The slope of L-FABP becomes − 0.03 and that of IL-6 − 0.029 as time passes from onset, indicating that the value of both L-FABP and IL-6 decreases overtime. In severe cases, the value of L-FABP tends to be high soon after the onset of symptoms, and to remain high until at least 15th day. Regarding groups, there is a significant difference in L-FABP to discriminate severe cases (*p* < 0.0001), but IL-6 was *p* = 0.344 and no significance. This suggest that L-FABP is more suitable to discriminate severe cases

Dynamics of L-FABP (*n* = 228) and IL-6 (*n* = 60) were evaluated in all subjects in whom both indicators were measured from onset to 15 days later (Fig. 7). As time passes from onset, the slope becomes − 0.03 for L-FABP and − 0.029 for IL-6. This shows that the values of both L-FABP and IL-6 decrease overtime. In severe cases, the value of L-FABP tends to be high soon after the onset of symptoms, and to remain high until at least 15th day. On the other hand, the value for moderate and mild remain low. The difference in severe cases was remarkable in L-FABP, and the mixed-effect model found a significant difference (*p* < 0.0001) to moderate or mild cases. Regarding IL-6, severe cases tended to show higher values in severe cases, but the difference was not significant to moderate or mild. Fewer cases and limited time points for serum collection in IL-6 might affect to statistical insignificance. IL-6 was reportedly high in severe cases around 10 days from onset. However, there are some severe cases showing the decrease of IL-6 level. It seems impossible to conclude in this analysis that IL-6 will take high value around 10 days on average value basis. Even though IL-6 was approved by the FDA as an indicator to discriminate severe cases, it frequently fails to distinguish mild from moderate cases. L-FABP does so with much greater certainty during the first 15 days. These dynamics are more evident in the earlier phase of infection.

### Operations to increase the probability of success in clinical trials

Most drugs in clinical use for COVID-19 have been developed for other infections or diseases, and have been applied as viral RNA-dependent RNA polymerase inhibitors, rheumatoid arthritis drugs, lupus drugs, etc. Clinical trials for treatment of COVID-19 infection have not progressed well, despite the growing need of the public. Especially in early stages, clinical trials faced difficulties caused by enrolling a large number of cases that would recover without medical intervention. It is important to note here that drugs with a solid companion diagnosis, such as tocilizumab, were developed quickly. If therapeutic intervention is not directed toward high-risk patients, drug development will be extremely difficult in infectious diseases in which spontaneous resolution is possible. As mentioned above, urinary L-FABP is an in vitro diagnostic agent that can serve as a companion diagnostic tool.

## Conclusion

Early risk assessment, where COVID-19 infection clusters occur, can facilitate subsequent management for both the government and patients. We developed a point-of-care test (POCT) using immuno-lateral flow of L-FABP and recently reported that the risk of severe disease in mild COVID-19 positive patients can be detected with 88.9% accuracy (sensitivity 100%, specificity 87.7%) by ROC analysis [10]. This has attracted much interest, especially in Europe and the United States, where self-care using OTC home tests is advancing. Although we have discussed COVID-19 here, this urinary L-FABP approach will be efficacious not only for COVID-19 cases, but also for SIRS and MARS, viral infections that display similar pathologies. Therefore, we expect this to be a useful risk management tool against future, unknown viral respiratory infections.

## Data Availability

Not applicable because it is a review article.
